# Bivalent mRNA-1273.214 vaccine effectiveness against SARS-CoV-2 omicron XBB^*^ infections

**DOI:** 10.1093/jtm/taad106

**Published:** 2023-08-09

**Authors:** Hiam Chemaitelly, Houssein H Ayoub, Sawsan AlMukdad, Jeremy S Faust, Patrick Tang, Peter Coyle, Hadi M Yassine, Asmaa A Al Thani, Hebah A Al-Khatib, Mohammad R Hasan, Zaina Al-Kanaani, Einas Al-Kuwari, Andrew Jeremijenko, Anvar H Kaleeckal, Ali N Latif, Riyazuddin M Shaik, Hanan F Abdul-Rahim, Gheyath K Nasrallah, Mohamed G Al-Kuwari, Adeel A Butt, Hamad E Al-Romaihi, Mohamed H Al-Thani, Abdullatif Al-Khal, Roberto Bertollini, Laith J Abu-Raddad

**Affiliations:** Infectious Disease Epidemiology Group, Weill Cornell Medicine-Qatar, Cornell University, PO Box 24144, Doha, Qatar; Departments of Mathematics, Statistics, and Physics, and of Biomedical Science, and of Public Health, Qatar University, PO Box 2713, Doha, Qatar; Infectious Disease Epidemiology Group, Weill Cornell Medicine-Qatar, Cornell University, PO Box 24144, Doha, Qatar; Department of Emergency Medicine, Brigham and Women’s Hospital, MA 02115, Boston, Massachusetts, USA; Department of Pathology, Sidra Medicine, PO Box 26999, Doha; Hamad Medical Corporation, POBox 3050, Doha, Qatar; Departments of Mathematics, Statistics, and Physics, and of Biomedical Science, and of Public Health, Qatar University, PO Box 2713, Doha, Qatar; Departments of Mathematics, Statistics, and Physics, and of Biomedical Science, and of Public Health, Qatar University, PO Box 2713, Doha, Qatar; Departments of Mathematics, Statistics, and Physics, and of Biomedical Science, and of Public Health, Qatar University, PO Box 2713, Doha, Qatar; Department of Pathology, Sidra Medicine, PO Box 26999, Doha; Hamad Medical Corporation, POBox 3050, Doha, Qatar; Hamad Medical Corporation, POBox 3050, Doha, Qatar; Hamad Medical Corporation, POBox 3050, Doha, Qatar; Hamad Medical Corporation, POBox 3050, Doha, Qatar; Hamad Medical Corporation, POBox 3050, Doha, Qatar; Hamad Medical Corporation, POBox 3050, Doha, Qatar; Departments of Mathematics, Statistics, and Physics, and of Biomedical Science, and of Public Health, Qatar University, PO Box 2713, Doha, Qatar; Departments of Mathematics, Statistics, and Physics, and of Biomedical Science, and of Public Health, Qatar University, PO Box 2713, Doha, Qatar; Primary Health Care Corporation, PO Box 26555, Doha, Qatar; Hamad Medical Corporation, POBox 3050, Doha, Qatar; Ministry of Public Health, PO Box 7744, doha, Qatar; Ministry of Public Health, PO Box 7744, doha, Qatar; Hamad Medical Corporation, POBox 3050, Doha, Qatar; Ministry of Public Health, PO Box 7744, doha, Qatar; Infectious Disease Epidemiology Group, Weill Cornell Medicine-Qatar, Cornell University, PO Box 24144, Doha, Qatar

**Keywords:** Hybrid immunity, natural infection, waning vaccine effectiveness, asymptomatic infection, Qatar, variant-containing COVID-19 vaccines

## Abstract

Effectiveness of the 50-μg mRNA-1273.214 bivalent vaccine against SARS-CoV-2 infection was modest at 25% in a matched, retrospective, cohort study in Qatar comparing infection incidence in the bivalent cohort to that in the national no-recent-vaccination resident cohort. XBB* immune evasion, immune imprinting effects, or both, may explain findings.

In October of 2022, Qatar introduced COVID-19 bivalent vaccination for persons ≥ 12 years using the 50-μg mRNA-1273.214 vaccine combining SARS-CoV-2 ancestral and omicron BA.1 strains.^1^ We estimated this vaccine’s effectiveness against SARS-CoV-2 infection.

Using Qatar’s national SARS-CoV-2 databases, we conducted a matched, retrospective, cohort study to compare infection incidence in the national cohort of persons who received the vaccine (bivalent cohort) to that in the national cohort of Qatar residents whose last vaccination was ≥6 months before follow-up start (no-recent-vaccination cohort; Supplementary Appendix 1). The 6-month cut-off was chosen because of negligible effectiveness of first-generation vaccines against omicron infection ≥ 6 months after vaccination.^2^

Incidence of infection was defined as the first SARS-CoV-2 PCR-positive or rapid-antigen-positive test after the start of follow-up, regardless of symptoms. Cohorts were balanced on observed confounders through exact matching. Follow-up started 7 days after the person in the bivalent cohort received their vaccine dose. Associations were estimated using Cox proportional-hazards models adjusted for the matching factors and testing rate.

During follow-up, 65 infections were recorded in the bivalent cohort and 406 in the no-recent-vaccination cohort. Cumulative incidence was 0.80% (95% CI: 0.61–1.07%) in the bivalent cohort and 1.00% (95% CI: 0.89–1.11%) in the no-recent-vaccination cohort, 150 days after follow-up start (Figure 1A). Incidence was dominated by omicron XBB* subvariants including XBB, XBB.1, XBB.1.5, XBB.1.9.1, XBB.1.9.2, XBB.1.16 and XBB.2.3.

**Figure 1:**
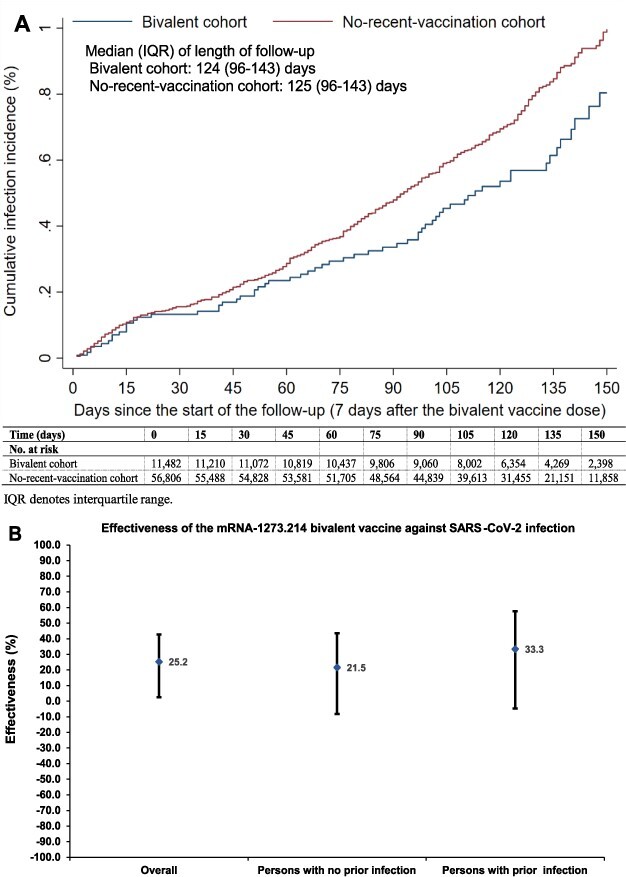
(A) Cumulative incidence of SARS-CoV-2 infection in the matched bivalent cohort and the no-recent-vaccination cohort. (B) mRNA-1273.214 bivalent vaccine effectiveness against SARS-CoV-2 infection overall and by documented prior infection status. Cohorts were matched exactly one-to-five by sex, 10-year age group, nationality, number of coexisting conditions and documented prior infection status to balance observed confounders between exposure groups.

Adjusted hazard ratio comparing infection incidence in the bivalent cohort to that in the no-recent-vaccination cohort was 0.75 (95% CI: 0.57–0.97; Figure 1B). Bivalent vaccine effectiveness was 25.2% (95% CI: 2.6–42.6%). Effectiveness was 21.5% (95% CI: −8.2 to 43.5%) amongst persons with no prior infection and 33.3% (95% CI: −4.6 to 57.6%) amongst persons with prior infection. In absence of severe, critical or fatal COVID-19 cases, effectiveness against severity could not be estimated.

Of matched individuals, 672 in the bivalent cohort (5.9%) and 3029 (5.3%) in the no-recent-vaccination cohort had a SARS-CoV-2 test during follow-up. Total number of tests was 855 in 3684.5 person-years and 3923 tests in 18 242.6 person-years, respectively. Testing frequency was 0.06 tests per person and 0.05 tests per person, respectively. Testing rate was 0.23 tests per person-year and 0.22 tests per person-year, respectively.

mRNA-1273.214 reduced incidence of SARS-CoV-2 infection, but the protection was modest at only 25%, consistent with a modest protection for bivalent vaccines combining ancestral and omicron BA.1 or BA.4/BA.5 strains.^3^^,^^4^ The modest protection may have risen because of XBB* immune evasion or immune imprinting effects,^2^^,^^5^ or combination of both. The modest protection might also be attributed to the bivalent vaccine’s limited ability to provide additional protection compared with the immunity developed through recent undocumented infections in both groups.^6^^,^^7^

This study has limitations. With the relatively young population of Qatar, findings may not be generalizable to countries with a larger proportion of elderly citizens. Effectiveness was estimated by prior infection status, but some infections may have never been documented, suggesting potential for misclassification bias in defining prior-infection subgroups. Although no severe cases were recorded in this study, it is important to note that SARS-CoV-2 infection can lead to severe disease in certain subpopulations.^2^^,^^8^ Vaccination remains critical to prevent severe COVID-19 within these vulnerable groups.^2^^,^^9^ In conclusion, our findings highlight the need for a new generation of vaccines that offer effective, long-term protection against a broad spectrum of potential variants.

## Funding

We acknowledge the many dedicated individuals at Hamad Medical Corporation, the Ministry of Public Health, the Primary Health Care Corporation, the Qatar Biobank, Sidra Medicine and Weill Cornell Medicine—Qatar for their diligent efforts and contributions to make this study possible. The authors are grateful for support from the Biomedical Research Program and the Biostatistics, Epidemiology, and Biomathematics Research Core, both at Weill Cornell Medicine-Qatar, as well as for support provided by the Ministry of Public Health, Hamad Medical Corporation and Sidra Medicine. The authors are also grateful for the Qatar Genome Programme and Qatar University Biomedical Research Center for institutional support for the reagents needed for the viral genome sequencing. Statements made herein are solely the responsibility of the authors. The funders of the study had no role in study design, data collection, data analysis, data interpretation or writing of the article.

*Conflict of interest* Dr A.A.B. has received institutional grant funding from Gilead Sciences unrelated to the work presented in this paper. Otherwise we declare no competing interests.

## Authors’ contribution

H.C. co-designed the study, performed the statistical analyses and co-wrote the first draft of the article. L.J.A. conceived and co-designed the study, led the statistical analyses and co-wrote the first draft of the article. P.V.C. conducted viral genome sequencing and designed mass PCR testing to allow routine capture of SGTF variants. P.T. and M.R.H. conducted the multiplex, real-time reverse-transcription PCR variant screening and viral genome sequencing. H.Y., A.A.A-T. and H.A.K. conducted viral genome sequencing. All authors contributed to data collection and acquisition, database development, discussion and interpretation of the results, and to the writing of the manuscript. All authors have read and approved the final manuscript.

## Oversight and patients’ consent

The institutional review boards at Hamad Medical Corporation and Weill Cornell Medicine–Qatar approved this retrospective study with a waiver of informed consent.

## Data availability

The dataset of this study is a property of the Qatar Ministry of Public Health that was provided to the researchers through a restricted-access agreement that prevents sharing the dataset with a third party or publicly. The data are available under restricted access for preservation of confidentiality of patient data. Access can be obtained through a direct application for data access to Her Excellency the Minister of Public Health (https://www.moph.gov.qa/english/OurServices/eservices/Pages/Governmental-Health-Communication-Center.aspx). The raw data are protected and are not available due to data privacy laws. Data were available to authors through .csv files where information has been downloaded from the CERNER database system (no links/accession codes were available to authors). Aggregate data are available within the manuscript and its Supplementary appendix.

## Supplementary Material

Supplementary_Appendix_taad106
