# Supplementary material for: Bivalent mRNA-1273.214 vaccine effectiveness against SARS-CoV-2 omicron XBB* infections
Source: J Travel Med. 2023 Aug 9;30(5):taad106. doi: 10.1093/jtm/taad106 (PMC10481416; doi:10.1093/jtm/taad106)
Supplement: Supplementary_Appendix_taad106 [file supplementary_appendix_taad106.docx]

**Appendix**

**Table of Contents**

[**Study population and data sources** 2](#_Toc138944529)

[**Study design and cohorts** 5](#_Toc138944530)

[**Cohorts matching and follow-up** 6](#_Toc138944531)

[**Comorbidity classification** 8](#_Toc138944532)

[**Oversight** 9](#_Toc138944533)

[**Statistical analysis** 9](#_Toc138944534)

[**Limitations** 11](#_Toc138944535)

[Section S2. Laboratory methods and variant ascertainment 14](#_Toc138944536)

[**Real-time reverse-transcription polymerase chain reaction testing** 14](#_Toc138944537)

[**Rapid antigen testing** 14](#_Toc138944538)

[**Classification of infections by variant type** 15](#_Toc138944539)

[Section S3. COVID-19 severity, criticality, and fatality classification 16](#_Toc138944540)

[Table S1. Baseline characteristics of eligible and matched cohorts of those who received the 50-μg bivalent mRNA-1273.214 vaccine (bivalent cohort) and those with no COVID-19 vaccination record in the past six months (no-recent-vaccination cohort). 18](#_Toc138944541)

[Figure S1. Cohort selection for investigating effectiveness of the 50-μg bivalent mRNA-1273.214 vaccine relative to that of no vaccination in the past six months in Qatar. 19](#_Toc138944542)

[Table S2. STROBE checklist for cohort studies. 20](#_Toc138944543)

[References 22](#_Toc138944544)

#

**Section S1.** **Detailed study methods**

## **Study population and data sources**

This study was conducted on the population of Qatar including data between October 18, 2022, the earliest bivalent vaccination record, and May 21, 2023. It analyzed the national, federated databases for coronavirus disease 2019 (COVID-19) laboratory testing, vaccination, hospitalization, and death, retrieved from the integrated, nationwide, digital-health information platform. Databases include all severe acute respiratory syndrome coronavirus 2 (SARS-CoV-2)-related data with no missing information since the onset of the pandemic, including all polymerase chain reaction (PCR) tests and medically supervised rapid antigen tests (Section S2).

Qatar’s national and universal public healthcare system uses the Cerner-system advanced digital health platform to track all electronic health record encounters of each individual in the country, including all citizens and residents registered in the national and universal public healthcare system. Registration in the public healthcare system is mandatory for citizens and residents.

The databases analyzed in this study are data-extract downloads from the Cerner-system that have been implemented on a regular (twice weekly) schedule since the onset of pandemic by the Business Intelligence Unit at Hamad Medical Corporation. Hamad Medical Corporation is the national public healthcare provider in Qatar. At every download all tests, COVID-19 vaccinations, hospitalizations related to COVID-19, and all death records regardless of cause are provided to the authors through .csv files. These databases have been analyzed throughout the pandemic not only for study-related purposes, but also to provide policymakers with summary data and analytics to inform the national response.

Every health encounter in the Cerner-system is linked to a unique individual through the HMC Number that links all records for this individual at the national level. Databases were merged and analyzed using the HMC Number to link all records whether for testing, vaccinations, hospitalizations, and deaths. All deaths in Qatar are tracked by the public healthcare system. All COVID-19-related healthcare was provided only in the public healthcare system. No private entity was permitted to provide COVID-19-related hospitalization. COVID-19 vaccination was also provided only through the public healthcare system. These health records were tracked throughout the COVID-19 pandemic using the Cerner system. This system has been implemented in 2013, before the onset of the pandemic. Therefore, we had all health records related to this study for the full national cohort of citizens and residents throughout the pandemic. This allowed us to follow each person over time.

Demographic details for every HMC Number (individual) such as sex, age, and nationality are collected upon issuing of the universal health card, based on the Qatar Identity Card, which is a mandatory requirement by the Ministry of Interior to every citizen and resident in the country. Date of expiry of Qatar Identity Card is collected and updated at encounters with the public healthcare system. Data extraction from the Qatar Identity Card to the digital health platform is performed electronically through scanning techniques.

All SARS-CoV-2 testing in any facility in Qatar is tracked nationally in one database, the national testing database. This database covers all testing in all locations and facilities throughout the country, whether public or private. Every PCR test and a proportion of the facility-based rapid antigen tests conducted in Qatar, regardless of location or setting, are classified on the basis of symptoms and the reason for testing (clinical symptoms, contact tracing, surveys or random testing campaigns, individual requests, routine healthcare testing, pre-travel, at port of entry, or other).

Before November 1, 2022, SARS-CoV-2 testing in Qatar was done at a mass scale where close to 5% of the population were tested every week.^1, 2^ Based on the distribution of the reason for testing up to November 1, 2022, most of the tests in Qatar were conducted for routine reasons, such as being travel-related, and about 75% of cases were diagnosed not because of appearance of symptoms, but because of routine testing.^1, 2^

Starting from November 1, 2022, SARS-CoV-2 testing was substantially reduced, but still close to 1% of the population are tested every week.^1, 2^ The distribution of the reason for testing during the study period, that is between October 18, 2022 and May 21, 2023, showed that 58.9% of all tests were conducted for routine reasons. However, only 37.5% of infections were diagnosed because of routine testing. All testing results in the national testing database during follow-up in the present study were factored in the analyses of this study.

The first large omicron wave that peaked in January of 2022 was massive and strained the testing capacity in the country.^1, 3, 4^ Accordingly, rapid antigen testing was introduced to relieve the pressure on PCR testing. Implementation of this change in testing occurred quickly precluding incorporation of reason for testing in large proportion of the rapid antigen tests for several months. While the reason for testing is available for all PCR tests, it is not available for all rapid antigen tests. Availability of reason for testing for the rapid antigen tests also varied with time.

Rapid antigen test kits are available for purchase in pharmacies in Qatar, but outcome of home-based testing is not reported nor documented in the national databases. Since SARS-CoV-2-test outcomes are linked to specific public health measures, restrictions, and privileges, testing policy and guidelines stress facility-based testing as the core testing mechanism in the population. While facility-based testing is provided free of charge or at low subsidized costs, depending on the reason for testing, home-based rapid antigen testing is de-emphasized and not supported as part of national policy. We are not aware of a reason to believe that home-based testing could have differentially affected the followed matched cohorts to affect our results.

Qatar has unusually young, diverse demographics, in that only 9% of its residents are ≥50 years of age, and 89% are expatriates from over 150 countries.^5, 6^ Qatar launched its COVID-19 vaccination program in December of 2020 using BNT162b2 and mRNA-1273 vaccines,^7^ and initiated vaccination with the 50-μg bivalent mRNA-1273.214 vaccine^8^ in October of 2022. These vaccines are accessible at multiple facilities throughout the country and are provided free charge regardless of citizenship or residency status. Further descriptions of the study population and these national databases were reported previously.^1, 2, 6, 9, 10^

## **Study design and cohorts**

We conducted an observational, matched, retrospective, cohort study that emulated a randomized “target” trial.^10, 11^ Incidence of breakthrough infection and associated severe, critical, or fatal COVID-19 were compared in the national cohort of persons who received a bivalent vaccine dose (designated the bivalent cohort) to that in the national cohort of Qatar residents who received their last vaccine dose at least 6 months before the start of the follow-up (designated the no-recent-vaccination cohort). The 6-month vaccination cut-off was chosen because effectiveness against infection of the original BNT162b2 and mRNA-1273 vaccines, which are based on the ancestral SARS-CoV-2 virus, is negligible at the seventh month and thereafter after the vaccine dose.^4, 12^ This cohort thus provides an appropriate comparator cohort to assess effectiveness of the bivalent vaccine.

Incidence of infection was defined as the first PCR-positive or rapid-antigen-positive test after the start of follow-up, regardless of symptoms. Infection severity classification followed World Health Organization guidelines for COVID-19 case severity (acute-care hospitalizations),^13^ criticality (intensive-care-unit hospitalizations),^13^ and fatality^14^ (Section S3).

## **Cohorts matching and follow-up**

Cohorts were matched exactly one-to-five by sex, 10-year age group, nationality, number of coexisting conditions (0, 1, 2, 3, 4, 5, or ≥6 coexisting conditions), and prior infection status (no prior infection, or prior infection with either pre-omicron or omicron viruses, or prior infections with both viruses) to balance observed confounders between exposure groups that are related to risk of infection.^6, 15-18^ Prior infections were classified as pre-omicron if they occurred before December 19, 2021, the date of onset of the omicron wave in Qatar,^3^ and as omicron otherwise. Matching by the considered factors was informed by results of prior studies that used matching to control for differences in infection exposure risk in Qatar.^2, 7, 19-21^

Persons were eligible for inclusion in the bivalent cohort if they received a dose of the 50-μg mRNA-1273.214 bivalent vaccine (25 μg each of ancestral Wuhan-Hu-1 and omicron B.1.1.529 [BA.1] spike messenger RNAs)^8^ and had no record for a SARS-CoV-2-positive test within 90 days before the start of follow-up. The latter exclusion criterion, applied to both arms of the study, ensured that infections after start of follow-up were incident infections and not prolonged SARS-CoV-2-positivity of earlier infections.^3, 22, 23^ Any person with an active residency status in Qatar and a record for SARS-CoV-2 testing in the national database was eligible for inclusion in the no-recent-vaccination cohort.

Matching was performed iteratively such that persons in the no-recent-vaccination cohort had, at the start of the follow-up, an active residency permit, the same prior infection status as their match, and no record for a vaccine dose in the past six months, or for a SARS-CoV-2-positive test in the previous 90 days. Controls that did not fulfil these criteria were dropped and replaced by other eligible controls. The matching algorithm was implemented using *ccmatch* command in Stata supplemented with conditions to retain only controls that fulfil the eligibility criteria and was iterated using loops with as many replications as needed until exhaustion (i.e., no more matched pairs could be identified).

Since this study was designed to emulate a target trial,^10, 11^ the matching algorithm was developed and exact matching was used to ensure that both cohorts are similar in terms of all factors known, or have any potential to affect, risk of infection, other than the bivalent vaccine effect. Informed by prior epidemiologic studies on this population, including established associations with infection,^6, 15-18^ as well the possibility that prior infection status may affect vaccine effectiveness, matching was done to control for any differences in risk of infection between cohorts, for the same purpose as that of randomization in a randomized controlled trial. Just as randomized controlled trials select a sample of the national population that fits eligibility criteria, this study selected a sample of the national population conditional on an exact balance of observed confounders between study arms. Yet, despite the strict matching, the study matched sample is several folds larger than a typical COVID-19 vaccine randomized controlled trial.

Persons in the matched no-recent-vaccination cohort contributed follow-up time in the study before receiving the bivalent dose (while matched to persons in the bivalent cohort), and subsequently contributed follow-up time in the bivalent cohort, if they received a bivalent dose (while matched to persons in the no-recent-vaccination cohort). Introducing this cross-over in the study design provides a basis for separating vaccination effects from other effects and may reduce potential differences arising from unmeasured behaviors.

As in previous studies,^10, 24^ to ensure time for sufficient immunogenicity, both members of each matched pair were followed starting 7 days after the calendar date in which the person in the bivalent cohort received their vaccine dose. For exchangeability,^10, 24^ both members of each matched pair were censored at earliest occurrence of a person receiving a new vaccine dose. Accordingly, individuals were followed up until the first of any of the following events: a documented SARS-CoV-2 infection (regardless of symptoms), or a new vaccine dose for persons in the bivalent cohort (with matched-pair censoring) or for persons in the no-recent-vaccination cohort (with matched-pair censoring), or death, or administrative end of follow-up (April 5, 2023).

## **Comorbidity classification**

Comorbidities were ascertained and classified based on the ICD-10 codes as recorded in the electronic health record encounters of each individual in the Cerner-system national database that includes all citizens and residents registered in the national and universal public healthcare system. The public healthcare system provides healthcare to the entire resident population of Qatar free of charge or at heavily subsidized costs, including prescription drugs.

All encounters for each individual were analyzed to determine the comorbidity classification for that individual, as part of a recent national analysis to assess healthcare needs and resource allocation. The Cerner-system national database includes encounters starting from 2013, after this system was launched in Qatar. As long as each individual had at least one encounter with a specific comorbidity diagnosis since 2013, this person was classified with this comorbidity.

Individuals who have comorbidities but never sought care in the public healthcare system, or seek care exclusively in private healthcare facilities, were classified as individuals with no comorbidity due to absence of recorded encounters for them.

## **Oversight**

The institutional review boards at Hamad Medical Corporation and Weill Cornell Medicine–Qatar approved this retrospective study with a waiver of informed consent. The study was reported according to the Strengthening the Reporting of Observational Studies in Epidemiology (STROBE) guidelines (Table S2). The authors vouch for the accuracy and completeness of the data and for the fidelity of the study to the protocol. Data used in this study are the property of the Ministry of Public Health of Qatar and were provided to the researchers through a restricted-access agreement for preservation of confidentiality of patient data. The funders had no role in the study design; the collection, analysis, or interpretation of the data; or the writing of the manuscript.

## **Statistical analysis**

Eligible and matched cohorts were described using frequency distributions and measures of central tendency and were compared using standardized mean differences (SMDs). An SMD of ≤0.1 indicated adequate matching. Cumulative incidence of infection (defined as proportion of persons at risk, whose primary endpoint during follow-up was an infection) was estimated using the Kaplan-Meier estimator method. Incidence rate of infection in each cohort, defined as number of identified infections divided by number of person-weeks contributed by all individuals in the cohort, was estimated, with the corresponding 95% confidence interval (CI) using a Poisson log-likelihood regression model with the Stata 17.0 *stptime* command.

The overall hazard ratio (HR), comparing incidence of infection in the cohorts and corresponding 95% CIs, was calculated using Cox regression adjusted for the matching factors with the Stata 17.0 *stcox* command. The adjustment for the matching factors was done to ensure precise and unbiased standard variance.^25^ The overall HR was further adjusted in the Cox regression for differences in testing rate (low testers, intermediate testers, and high testers defined as persons having ≤1, 2-6, and >6 tests per person-year during follow-up, respectively). This adjustment was done because a substantial proportion of SARS-CoV-2 testing in Qatar is done for routine reasons and not because of symptoms, thereby potentially introducing differential ascertainment of infection across the cohorts if routine testing varied by cohort. Schoenfeld residuals and log-log plots for survival curves were used to investigate the proportional-hazards assumption and to investigate its adequacy. Although the proportional-hazards assumption was not violated, the overall HR can be considered a weighted average of potential time-varying HR.^26^ 95% CIs were not adjusted for multiplicity; thus, they should not be used to infer definitive differences between groups. Interactions were not considered.

Vaccine effectiveness was estimated as 1-adjusted hazard ratio (aHR) if the aHR was <1, and as 1/aHR-1 if the aHR was ≥1.^4, 27^ The latter was to ensure symmetric scale for both negative and positive effectiveness, ranging from -100%-100%, leading to easier and meaningful interpretation of effectiveness, regardless of being positive or negative. For example, an effectiveness of 40% means that incidence in the bivalent cohort was 40% less than that in the no-recent-vaccination cohort. Meanwhile, an effectiveness of -40% means that incidence in the no-recent-vaccination cohort was 40% less than that in the bivalent cohort.

Subgroup analyses were conducted to estimate bivalent vaccine effectiveness by prior infection status. Statistical analyses were performed using Stata/SE version 17.0 (Stata Corporation, College Station, TX, USA).

## **Limitations**

This study has limitations. With the relatively young population of Qatar, our findings may not be generalizable to other countries where elderly citizens constitute a larger proportion of the total population. Also with the relatively young population of Qatar,^6, 28^ and the lower severity of omicron infections,^29-31^ there were no confirmed severe,^13^ critical,^13^ and fatal^14^ COVID-19 cases to estimate bivalent vaccine effectiveness against severe forms of COVID-19. The national cohort of persons receiving the bivalent dose was relatively small to allow estimation of hazard ratios month-by-month since the start of the follow-up.

Qatar has unusually diverse demographics in that 89% of the population are expatriates from over 150 countries.^6^ Data on travel history of the study population were not available. Since most of the population is an expatriate population, it is plausible that the rate of travel is higher than in other countries. Accordingly, an active residency requirement was added to ensure residency in Qatar of matched persons at study recruitment. Possibility of travel is also one of the reasons for matching by nationality, age, and sex; to balance rates of travel across the cohorts. These demographic factors provide a powerful proxy for socio-economic status and occupation in this country,^6, 16, 17^ and thus of the rate of travel outside the country.

Receiving a bivalent vaccine dose could be correlated with health-seeking behavior that could possibly be associated with more frequent testing, but testing rates were essentially identical in the matched cohorts. Home-based rapid-antigen testing is not documented and is not factored in these analyses. However, we are not aware of a reason to believe that home-based testing could have differentially affected the followed cohorts to alter study estimates. Matching was done while factoring key socio-demographic characteristics of the population,^6, 16, 17^ and this may also have controlled or reduced differences in home-based testing between cohorts.

Effectiveness was estimated by prior infection status, but some infections may have never been documented, thereby introducing the possibility of misclassification bias in defining some of the prior-infection subgroups, particularly the no-prior infection subgroup. The variant status of prior infections was determined by time of infection on the basis of the variant that was dominant at the time, and not based on viral genome sequencing of every infection. This may have introduced (a slight) misclassification bias in the variant status of prior infections.

As an observational study, investigated cohorts were neither blinded nor randomized, so unmeasured or uncontrolled confounding cannot be excluded. Although matching covered key factors affecting infection exposure,^6, 16, 17^ it was not possible for other factors such as geography or occupation, for which data were unavailable. However, Qatar is essentially a city state and infection incidence was broadly distributed across neighborhoods. Nationality, age, and sex, factors that were used in the matching, provide a powerful proxy for socio-economic status and occupation in this country.^6, 16, 17^

The matching procedure used in this study was investigated in previous studies of different epidemiologic designs, and using control groups to test for null effects.^2, 7, 19-21^ These control groups have included unvaccinated cohorts versus vaccinated cohorts within two weeks of the first dose^2, 19-21^ (when vaccine protection is negligible^32^), and mRNA-1273- versus BNT162b2-vaccinated cohorts, also in the first two weeks after the first dose.^7^ These prior studies demonstrated at different times during the pandemic that this procedure resulted in similar infection exposure levels across groups,^2, 7, 19-21^ suggesting that the matching strategy may also have controlled for differences in infection exposure in the present study. Analyses were implemented on Qatar’s total population, perhaps minimizing the likelihood of bias. Since this study emulated a target trial,^10, 11^ the matching algorithm was developed and exact matching was used to ensure that both cohorts are similar in terms of all factors known, or have any potential to affect, risk of infection, other than the bivalent vaccine effect. With nearly the full cohort retained after the matching, the matched cohort can be considered representative of the full cohort.

# **Section S2. Laboratory methods and variant ascertainment**

## **Real-time reverse-transcription polymerase chain reaction testing**

Nasopharyngeal and/or oropharyngeal swabs were collected for polymerase chain reaction (PCR) testing and placed in Universal Transport Medium (UTM). Aliquots of UTM were: 1) extracted on KingFisher Flex (Thermo Fisher Scientific, USA), MGISP-960 (MGI, China), or ExiPrep 96 Lite (Bioneer, South Korea) followed by testing with real-time reverse-transcription PCR (RT-qPCR) using TaqPath COVID-19 Combo Kits (Thermo Fisher Scientific, USA) on an ABI 7500 FAST (Thermo Fisher Scientific, USA); 2) tested directly on the Cepheid GeneXpert system using the Xpert Xpress SARS-CoV-2 (Cepheid, USA); or 3) loaded directly into a Roche cobas 6800 system and assayed with the cobas SARS-CoV-2 Test (Roche, Switzerland). The first assay targets the viral S, N, and ORF1ab gene regions. The second targets the viral N and E-gene regions, and the third targets the ORF1ab and E-gene regions.

All PCR testing was conducted at the Hamad Medical Corporation Central Laboratory or Sidra Medicine Laboratory, following standardized protocols.

## **Rapid antigen testing**

Severe acute respiratory syndrome coronavirus 2 (SARS-CoV-2) antigen tests were performed on nasopharyngeal swabs using one of the following lateral flow antigen tests: Panbio COVID-19 Ag Rapid Test Device (Abbott, USA); SARS-CoV-2 Rapid Antigen Test (Roche, Switzerland); Standard Q COVID-19 Antigen Test (SD Biosensor, Korea); or CareStart COVID-19 Antigen Test (Access Bio, USA). All antigen tests were performed point-of-care according to each manufacturer’s instructions at public or private hospitals and clinics throughout Qatar with prior authorization and training by the Ministry of Public Health (MOPH). Antigen test results were electronically reported to the MOPH in real time using the Antigen Test Management System which is integrated with the national Coronavirus Disease 2019 (COVID-19) database.

## **Classification of infections by variant type**

Surveillance for SARS-CoV-2 variants in Qatar is based on viral genome sequencing and multiplex RT-qPCR variant screening^33^ of random positive clinical samples,^2, 20, 34-37^ complemented by deep sequencing of wastewater samples.^35, 38, 39^ Further details on the viral genome sequencing and multiplex RT-qPCR variant screening throughout the SARS-CoV-2 waves in Qatar can be found in previous publications.^1-3, 10, 12, 20, 34-37, 40-43^

# **Section S3. COVID-19 severity, criticality, and fatality classification**

Classification of Coronavirus Disease 2019 (COVID-19) case severity (acute-care hospitalizations),^13^criticality (intensive-care-unit hospitalizations),^13^ and fatality^14^ followed World Health Organization (WHO) guidelines. Assessments were made by trained medical personnel independent of study investigators and using individual chart reviews, as part of a national protocol applied to every hospitalized COVID-19 patient. Each hospitalized COVID-19 patient underwent an infection severity assessment every three days until discharge or death. We classified individuals who progressed to severe, critical, or fatal COVID-19 between the time of the documented infection and the end of the study based on their worst outcome, starting with death,^14^ followed by critical disease,^13^ and then severe disease.^13^

Severe COVID-19 disease was defined per WHO classification as a SARS-CoV-2 infected person with “oxygen saturation of <90% on room air, and/or respiratory rate of >30 breaths/minute in adults and children >5 years old (or ≥60 breaths/minute in children <2 months old or ≥50 breaths/minute in children 2-11 months old or ≥40 breaths/minute in children 1–5 years old), and/or signs of severe respiratory distress (accessory muscle use and inability to complete full sentences, and, in children, very severe chest wall indrawing, grunting, central cyanosis, or presence of any other general danger signs)”.^13^ Detailed WHO criteria for classifying Severe acute respiratory syndrome coronavirus 2 (SARS-CoV-2) infection severity can be found in the WHO technical report.^13^

Critical COVID-19 disease was defined per WHO classification as a SARS-CoV-2 infected person with “acute respiratory distress syndrome, sepsis, septic shock, or other conditions that would normally require the provision of life sustaining therapies such as mechanical ventilation (invasive or non-invasive) or vasopressor therapy”.^13^ Detailed WHO criteria for classifying SARS-CoV-2 infection criticality can be found in the WHO technical report.^13^

COVID-19 death was defined per WHO classification as “a death resulting from a clinically compatible illness, in a probable or confirmed COVID-19 case, unless there is a clear alternative cause of death that cannot be related to COVID-19 disease (e.g. trauma). There should be no period of complete recovery from COVID-19 between illness and death. A death due to COVID-19 may not be attributed to another disease (e.g. cancer) and should be counted independently of preexisting conditions that are suspected of triggering a severe course of COVID-19”. Detailed WHO criteria for classifying COVID-19 death can be found in the WHO technical report.^14^

#

# **Table S1. Baseline characteristics of eligible and matched cohorts of those who received the 50-μg bivalent mRNA-1273.214 vaccine (bivalent cohort) and those with no COVID-19 vaccination record in the past six months (no-recent-vaccination cohort).**

| **Characteristics** | **Full eligible cohorts** | | | **Matched cohorts^*^** | | |
| --- | --- | --- | --- | --- | --- | --- |
|  | **Bivalent cohort** | **No-recent-vaccination cohort^†^** | **SMD^‡^** | **Bivalent cohort** | **No-recent-vaccination cohort^†^** | **SMD^‡^** |
|  | **N=11,520** | **N=3,178,483** |  | **N=****11,482** | **N=56,806** |  |
| Median age (IQR)—years | 36 (31-45) | 32 (24-40) | 0.54^§^ | 37 (31-45) | 36 (31-44) | 0.03^§^ |
| Age—years |  |  |  |  |  |  |
| 0-9 years | 0 (0) | 309,478 (9.7) | 0.61 | -- | -- | 0.01 |
| 10-19 years | 298 (2.6) | 233,422 (7.3) |  | 298 (2.6) | 1,463 (2.6) |  |
| 20-29 years | 2,000 (17.4) | 756,644 (23.8) |  | 1,999 (17.4) | 9,921 (17.5) |  |
| 30-39 years | 4,669 (40.5) | 1,042,925 (32.8) |  | 4,666 (40.6) | 23,255 (40.9) |  |
| 40-49 years | 2,659 (23.1) | 549,117 (17.3) |  | 2,650 (23.1) | 13,041 (23.0) |  |
| 50-59 years | 1,373 (11.9) | 210,496 (6.6) |  | 1,359 (11.8) | 6,686 (11.8) |  |
| 60-69 years | 441 (3.8) | 59,487 (1.9) |  | 438 (3.8) | 2,112 (3.7) |  |
| 70+ years | 80 (0.7) | 16,914 (0.5) |  | 72 (0.6) | 328 (0.6) |  |
| Sex |  |  |  |  |  |  |
| Male | 7,725 (67.1) | 2,254,274 (70.9) | 0.08 | 7,708 (67.1) | 38,274 (67.4) | 0.00 |
| Female | 3,795 (32.9) | 924,209 (29.1) |  | 3,774 (32.9) | 18,532 (32.6) |  |
| Nationality^¶^ |  |  |  |  |  |  |
| Bangladeshi | 2,571 (22.3) | 287,600 (9.0) | 0.75 | 2,569 (22.4) | 12,818 (22.6) | 0.02 |
| Egyptian | 295 (2.6) | 170,665 (5.4) |  | 295 (2.6) | 1,475 (2.6) |  |
| Filipino | 2,546 (22.1) | 261,113 (8.2) |  | 2,546 (22.2) | 12,696 (22.3) |  |
| Indian | 2,727 (23.7) | 823,589 (25.9) |  | 2,727 (23.7) | 13,611 (24.0) |  |
| Nepalese | 1,102 (9.6) | 341,031 (10.7) |  | 1,102 (9.6) | 5,498 (9.7) |  |
| Pakistani | 557 (4.8) | 172,719 (5.4) |  | 557 (4.8) | 2,770 (4.9) |  |
| Qatari | 101 (0.9) | 311,947 (9.8) |  | 101 (0.9) | 505 (0.9) |  |
| Sri Lankan | 308 (2.7) | 117,004 (3.7) |  | 308 (2.7) | 1,534 (2.7) |  |
| Sudanese | 46 (0.4) | 70,421 (2.2) |  | 46 (0.4) | 226 (0.4) |  |
| Other nationalities^**^ | 1,267 (11.0) | 622,394 (19.6) |  | 1,231 (10.7) | 5,673 (10.0) |  |
| Number of coexisting conditions |  |  |  |  |  |  |
| None | 9,570 (83.1) | 2,695,427 (84.8) | 0.09 | 9,564 (83.3) | 47,542 (83.7) | 0.01 |
| 1 | 931 (8.1) | 273,640 (8.6) |  | 922 (8.0) | 4,499 (7.9) |  |
| 2 | 493 (4.3) | 108,758 (3.4) |  | 482 (4.2) | 2,349 (4.1) |  |
| 3 | 255 (2.2) | 45,742 (1.4) |  | 252 (2.2) | 1,207 (2.1) |  |
| 4 | 115 (1.0) | 25,525 (0.8) |  | 110 (1.0) | 511 (0.9) |  |
| 5 | 88 (0.8) | 14,627 (0.5) |  | 86 (0.7) | 384 (0.7) |  |
| 6+ | 68 (0.6) | 14,764 (0.5) |  | 66 (0.6) | 314 (0.6) |  |
| Prior infection status^††^ |  |  |  |  |  |  |
| No prior infection | 8,193 (71.1) | -- | -- | 8,185 (71.3) | 40,707 (71.7) | 0.01 |
| Pre-omicron | 1,059 (9.2) | -- |  | 1,051 (9.1) | 5,172 (9.1) |  |
| Omicron | 2,045 (17.7) | -- |  | 2,028 (17.7) | 9,934 (17.5) |  |
| Pre-omicron & omicron | 223 (1.9) | -- |  | 218 (1.9) | 993 (1.7) |  |

COVID-19 denotes coronavirus disease 2019, IQR interquartile range, SARS-CoV-2 severe acute respiratory syndrome coronavirus 2, and SMD standardized mean difference.

^*^Individuals in the bivalent cohort were matched exactly one-to-five by sex, 10-year age group, nationality, and number of coexisting conditions to individuals in the no-recent-vaccination cohort who had, at the start of the follow-up, an active residency permit, the same prior infection status as their match, and no record for a vaccine dose in the past six months, or for a SARS-CoV-2-positive test in the previous 90 days.

^†^All persons in the bivalent cohort were part of the no-recent-vaccination cohort prior to receiving the bivalent vaccine.

^‡^SMD is the difference in the mean of a covariate between groups divided by the pooled standard deviation. An SMD ≤0.1 indicates adequate matching.

^§^SMD is for the mean difference between groups divided by the pooled standard deviation.

^¶^Nationalities were chosen to represent the most populous groups in Qatar.

^**^These comprise up to 187 other nationalities in the unmatched cohorts, and 89 other nationalities in the matched cohorts.

^††^Ascertained at the start of follow-up. Accordingly, distribution is not available for the unmatched no-recent-vaccination cohort, as the start of follow-up for each person in the no-recent-vaccination cohort is determined by that of their match in the bivalent cohort (7 days after the bivalent dose) after the matching is performed.

# **Figure S1. Cohort selection for investigating effectiveness of the 50-μg bivalent mRNA-1273.214 vaccine relative to that of no vaccination in the past six months in Qatar.**

**

**

# **Table S2. STROBE checklist for cohort studies.**

|  | Item No | Recommendation | Main Text page |
| --- | --- | --- | --- |
| **Title and abstract** | 1 | (*a*) Indicate the study’s design with a commonly used term in the title or the abstract | Main text, Page 2, Paragraph 2 |
|  |  | (*b*) Provide in the abstract an informative and balanced summary of what was done and what was found | Not applicable |
| Introduction | | | |
| Background/rationale | 2 | Explain the scientific background and rationale for the investigation being reported | Main text, Page 2, Paragraph 1 |
| Objectives | 3 | State specific objectives, including any prespecified hypotheses | Main text, Page 2, Paragraph 1 |
| Methods | | | |
| Study design | 4 | Present key elements of study design early in the paper | Main text, Page 2, Paragraphs 2-3 |
| Setting | 5 | Describe the setting, locations, and relevant dates, including periods of recruitment, exposure, follow-up, and data collection | Main text, Page 2, Paragraphs 2-3 & Section S1 (‘Study population and data sources’, ‘Study design and cohorts’, & ‘Cohorts matching and follow-up’) in Appendix |
| Participants | 6 | (*a*) Give the eligibility criteria, and the sources and methods of selection of participants. Describe methods of follow-up | Main text, Page 2, Paragraphs 2-3 & Section S1 (‘Study population and data sources’, ‘Study design and cohorts’, & ‘Cohorts matching and follow-up’) in Appendix |
|  |  | (*b*) For matched studies, give matching criteria and number of exposed and unexposed |  |
| Variables | 7 | Clearly define all outcomes, exposures, predictors, potential confounders, and effect modifiers. Give diagnostic criteria, if applicable | Main text, Page 2, Paragraphs 1-3, & Table S1 & Sections S1-S3 in Appendix |
| Data sources/ measurement | 8* | For each variable of interest, give sources of data and details of methods of assessment (measurement). Describe comparability of assessment methods if there is more than one group | Main text, Page 2, Paragraphs 2-3, & Table S1, Section S1 (‘Study population and data sources’, ‘Study design and cohorts’, ‘Cohorts matching and follow-up’, & ‘‘Statistical analysis’, paragraph 1’) & Sections S2-S3 in Appendix |
| Bias | 9 | Describe any efforts to address potential sources of bias | Main text, Page 2, Paragraphs 2-3, & Section S1 (‘Cohorts matching and follow-up’ & ‘Statistical analysis’) in Appendix |
| Study size | 10 | Explain how the study size was arrived at | Figure S1 in Appendix |
| Quantitative variables | 11 | Explain how quantitative variables were handled in the analyses. If applicable, describe which groupings were chosen and why | Table S1 & Section S1 (‘Cohort matching and follow-up’ & ‘Statistical analysis’) in Appendix |
| Statistical methods | 12 | (*a*) Describe all statistical methods, including those used to control for confounding | Section S1 (‘Statistical analysis’) in Appendix |
|  |  | (*b*) Describe any methods used to examine subgroups and interactions | Section S1 (‘Statistical analysis’) in Appendix |
|  |  | (*c*) Explain how missing data were addressed | Not applicable, see Section S1 (‘Study population and data sources’) in Appendix |
|  |  | (*d*) If applicable, explain how loss to follow-up was addressed | Not applicable, see Section S1 (‘Study population and data sources’) in Appendix |
|  |  | (*e*) Describe any sensitivity analyses | Not applicable |
| Results | | |  |
| Participants | 13* | (a) Report numbers of individuals at each stage of study—eg numbers potentially eligible, examined for eligibility, confirmed eligible, included in the study, completing follow-up, and analysed | Figure S1 in Appendix |
|  |  | (b) Give reasons for non-participation at each stage |  |
|  |  | (c) Consider use of a flow diagram |  |
| Descriptive data | 14 | (a) Give characteristics of study participants (eg demographic, clinical, social) and information on exposures and potential confounders | Main text, Page 2, Paragraph 4 & Table S1 in Appendix |
|  |  | (b) Indicate number of participants with missing data for each variable of interest | Not applicable, see Section S1 (‘Study population and data sources’) in Appendix |
|  |  | (c) Summarise follow-up time (eg, average and total amount) | Figure 1A |
| Outcome data | 15 | Report numbers of outcome events or summary measures over time | Main text, Page 2, Paragraph 5, Figure 1, & Table S1 & Figure S1 in Appendix |
| Main results | 16 | (a) Give unadjusted estimates and, if applicable, confounder-adjusted estimates and their precision (eg, 95% confidence interval). Make clear which confounders were adjusted for and why they were included | Main text, Page 3, Paragraph 2, & Figure 1B |
|  |  | (b) Report category boundaries when continuous variables were categorized | Table S1 in Appendix |
|  |  | (c) If relevant, consider translating estimates of relative risk into absolute risk for a meaningful time period | Not applicable |
| Other analyses | 17 | Report other analyses done—eg analyses of subgroups and interactions, and sensitivity analyses | Main text, Page 3, Paragraphs 2-3 |
| Discussion | | | |
| Key results | 18 | Summarise key results with reference to study objectives | Main text, Page 3, Paragraph 4 & Page 4, Paragraph 1 |
| Limitations | 19 | Discuss limitations of the study, taking into account sources of potential bias or imprecision. Discuss both direction and magnitude of any potential bias | Main text, Page 3, Paragraph 2 & Section S1 ‘Limitations’ in Supplement |
| Interpretation | 20 | Give a cautious overall interpretation of results considering objectives, limitations, multiplicity of analyses, results from similar studies, and other relevant evidence | Main text, Page 3, Paragraph 5 |
| Generalisability | 21 | Discuss the generalisability (external validity) of the study results | Main text, Page 3, Paragraph 5 |
| Other information | | | |
| Funding | 22 | Give the source of funding and the role of the funders for the present study and, if applicable, for the original study on which the present article is based | Funding |

# **References**

1 Altarawneh HN, Chemaitelly H, Ayoub HH, et al. Effects of previous infection and vaccination on symptomatic omicron infections. *N Engl J Med* 2022; **387**:21-34.

2 Chemaitelly H, Tang P, Hasan MR, et al. Waning of bnt162b2 vaccine protection against sars-cov-2 infection in qatar. *N Engl J Med* 2021; **385**:e83.

3 Altarawneh HN, Chemaitelly H, Hasan MR, et al. Protection against the omicron variant from previous sars-cov-2 infection. *N Engl J Med* 2022; **386**:1288-1290.

4 Chemaitelly H, Ayoub HH, Tang P, et al. Long-term covid-19 booster effectiveness by infection history and clinical vulnerability and immune imprinting: A retrospective population-based cohort study. *Lancet Infect Dis* 2023.

5 Planning and Statistics Authority-State of Qatar. Qatar monthly statistics. Available from: <Https://www.Psa.Gov.Qa/en/pages/default.Aspx>. Accessed on: May 26, 2020. 2020.

6 Abu-Raddad LJ, Chemaitelly H, Ayoub HH, et al. Characterizing the qatar advanced-phase sars-cov-2 epidemic. *Sci Rep* 2021; **11**:6233.

7 Abu-Raddad LJ, Chemaitelly H, Bertollini R, National Study Group for Covid Vaccination. Effectiveness of mrna-1273 and bnt162b2 vaccines in qatar. *N Engl J Med* 2022; **386**:799-800.

8 Chalkias S, Harper C, Vrbicky K, et al. A bivalent omicron-containing booster vaccine against covid-19. *New England Journal of Medicine* 2022; **387**:1279-1291.

9 Chemaitelly H, Bertollini R, Abu-Raddad LJ, National Study Group for Covid Epidemiology. Efficacy of natural immunity against sars-cov-2 reinfection with the beta variant. *N Engl J Med* 2021; **385**:2585-2586.

10 Abu-Raddad LJ, Chemaitelly H, Ayoub HH, et al. Effect of mrna vaccine boosters against sars-cov-2 omicron infection in qatar. *N Engl J Med* 2022; **386**:1804-1816.

11 Hernan MA, Robins JM. Using big data to emulate a target trial when a randomized trial is not available. *Am J Epidemiol* 2016; **183**:758-64.

12 Chemaitelly H, Ayoub HH, AlMukdad S, et al. Duration of mrna vaccine protection against sars-cov-2 omicron ba.1 and ba.2 subvariants in qatar. *Nat Commun* 2022; **13**:3082.

13 World Health Organization (WHO). Living guidance for clinical management of covid-19. Aavailable from: <Https://www.Who.Int/publications/i/item/who-2019-ncov-clinical-2021-2>. Accessed on: February 27, 2023. 2021.

14 World Health Organization (WHO). International guidelines for certification and classification (coding) of covid-19 as cause of death. Available from: <Https://www.Who.Int/publications/m/item/international-guidelines-for-certification-and-classification-(coding)-of-covid-19-as-cause-of-death>. Accessed on: February 27, 2023. 2020.

15 Ayoub HH, Chemaitelly H, Seedat S, et al. Mathematical modeling of the sars-cov-2 epidemic in qatar and its impact on the national response to covid-19. *J Glob Health* 2021; **11**:05005.

16 Coyle PV, Chemaitelly H, Ben Hadj Kacem MA, et al. Sars-cov-2 seroprevalence in the urban population of qatar: An analysis of antibody testing on a sample of 112,941 individuals. *iScience* 2021; **24**:102646.

17 Al-Thani MH, Farag E, Bertollini R, et al. Sars-cov-2 infection is at herd immunity in the majority segment of the population of qatar. *Open Forum Infect Dis* 2021; **8**:ofab221.

18 Jeremijenko A, Chemaitelly H, Ayoub HH, et al. Herd immunity against severe acute respiratory syndrome coronavirus 2 infection in 10 communities, qatar. *Emerg Infect Dis* 2021; **27**:1343-1352.

19 Abu-Raddad LJ, Chemaitelly H, Yassine HM, et al. Pfizer-biontech mrna bnt162b2 covid-19 vaccine protection against variants of concern after one versus two doses. *J Travel Med* 2021; **28**.

20 Chemaitelly H, Yassine HM, Benslimane FM, et al. Mrna-1273 covid-19 vaccine effectiveness against the b.1.1.7 and b.1.351 variants and severe covid-19 disease in qatar. *Nat Med* 2021; **27**:1614-1621.

21 Abu-Raddad LJ, Chemaitelly H, Bertollini R, National Study Group for Covid Vaccination. Waning mrna-1273 vaccine effectiveness against sars-cov-2 infection in qatar. *N Engl J Med* 2022; **386**:1091-1093.

22 Kojima N, Shrestha NK, Klausner JD. A systematic review of the protective effect of prior sars-cov-2 infection on repeat infection. *Eval Health Prof* 2021; **44**:327-332.

23 Pilz S, Theiler-Schwetz V, Trummer C, et al. Sars-cov-2 reinfections: Overview of efficacy and duration of natural and hybrid immunity. *Environ Res* 2022:112911.

24 Barda N, Dagan N, Cohen C, et al. Effectiveness of a third dose of the bnt162b2 mrna covid-19 vaccine for preventing severe outcomes in israel: An observational study. *Lancet* 2021; **398**:2093-2100.

25 Sjolander A, Greenland S. Ignoring the matching variables in cohort studies - when is it valid and why? *Stat Med* 2013; **32**:4696-708.

26 Stensrud MJ, Hernan MA. Why test for proportional hazards? *JAMA* 2020; **323**:1401-1402.

27 Tseng HF, Ackerson BK, Bruxvoort KJ, et al. Effectiveness of mrna-1273 against infection and covid-19 hospitalization with sars-cov-2 omicron subvariants: Ba.1, ba.2, ba.2.12.1, ba.4, and ba.5. *medRxiv* 2022:2022.09.30.22280573.

28 Seedat S, Chemaitelly H, Ayoub HH, et al. Sars-cov-2 infection hospitalization, severity, criticality, and fatality rates in qatar. *Sci Rep* 2021; **11**:18182.

29 Butt AA, Dargham SR, Loka S, et al. Covid-19 disease severity in children infected with the omicron variant. *Clin Infect Dis* 2022.

30 Butt AA, Dargham SR, Tang P, et al. Covid-19 disease severity in persons infected with the omicron variant compared with the delta variant in qatar. *J Glob Health* 2022; **12**:05032.

31 Butt AA, Dargham SR, Coyle P, et al. Covid-19 disease severity in persons infected with omicron ba.1 and ba.2 sublineages and association with vaccination status. *JAMA Intern Med* 2022.

32 Polack FP, Thomas SJ, Kitchin N, et al. Safety and efficacy of the bnt162b2 mrna covid-19 vaccine. *N Engl J Med* 2020; **383**:2603-2615.

33 Vogels C, Fauver J, Grubaugh N. Multiplexed rt-qpcr to screen for sars-cov-2 b.1.1.7, b.1.351, and p.1 variants of concern v.3. Dx.Doi.Org/10.17504/protocols.Io.Br9vm966. 2021.

34 Abu-Raddad LJ, Chemaitelly H, Butt AA, National Study Group for Covid Vaccination. Effectiveness of the bnt162b2 covid-19 vaccine against the b.1.1.7 and b.1.351 variants. *N Engl J Med* 2021; **385**:187-189.

35 National Project of Surveillance for Variants of Concern and Viral Genome Sequencing. Qatar viral genome sequencing data. Data on randomly collected samples. <Https://www.Gisaid.Org/phylodynamics/global/nextstrain/>. ed.^,eds., 2021.

36 Benslimane FM, Al Khatib HA, Al-Jamal O, et al. One year of sars-cov-2: Genomic characterization of covid-19 outbreak in qatar. *Front Cell Infect Microbiol* 2021; **11**:768883.

37 Hasan MR, Kalikiri MKR, Mirza F, et al. Real-time sars-cov-2 genotyping by high-throughput multiplex pcr reveals the epidemiology of the variants of concern in qatar. *Int J Infect Dis* 2021; **112**:52-54.

38 Saththasivam J, El-Malah SS, Gomez TA, et al. Covid-19 (sars-cov-2) outbreak monitoring using wastewater-based epidemiology in qatar. *Sci Total Environ* 2021; **774**:145608.

39 El-Malah SS, Saththasivam J, Jabbar KA, et al. Application of human rnase p normalization for the realistic estimation of sars-cov-2 viral load in wastewater: A perspective from qatar wastewater surveillance. *Environ Technol Innov* 2022; **27**:102775.

40 Tang P, Hasan MR, Chemaitelly H, et al. Bnt162b2 and mrna-1273 covid-19 vaccine effectiveness against the sars-cov-2 delta variant in qatar. *Nat Med* 2021; **27**:2136-2143.

41 Qassim SH, Chemaitelly H, Ayoub HH, et al. Effects of ba.1/ba.2 subvariant, vaccination and prior infection on infectiousness of sars-cov-2 omicron infections. *J Travel Med* 2022; **29**.

42 Altarawneh HN, Chemaitelly H, Ayoub HH, et al. Protective effect of previous sars-cov-2 infection against omicron ba.4 and ba.5 subvariants. *N Engl J Med* 2022; **387**:1620-1622.

43 Chemaitelly H, Tang P, Coyle P, et al. Protection against reinfection with the omicron ba.2.75 subvariant. *N Engl J Med* 2023; **388**:665-667.
